# Emergency management of shoulder fracture-dislocation—An assessment of current practice in the Emergency Department of a University Teaching Hospital

**DOI:** 10.1007/s11845-026-04351-w

**Published:** 2026-03-31

**Authors:** Joseph Slowey, Walter Cullen, John Broughan, John Ryan, Kieran O’Shea, Eric Heffernan

**Affiliations:** 1 St. Vincent’s University Hospital, Dublin, Ireland; 2 UCD School of Medicine, Dublin, Ireland

**Keywords:** Emergency department, Fracture dislocation, General anaesthesia, Greater tuberosity, Neck of humerus, Orthopaedic, Paralytic agent, Sedation, Shoulder, Theatre

## Abstract

**Background:**

Isolated Greater Tuberosity (GT) fracture dislocations are routinely reduced in the emergency department, but there is risk of missing an occult neck of humerus fracture in certain patients, unless a CT scan is performed prior to reduction Shaw et al (BMC Musculoskelet Disord 20:482, 2019). There is also a known risk of iatrogenic harm when reducing a dislocated shoulder involving a fractured neck of humerus and this risk is increased without the use of an anaesthetic paralysing agent Yuan et al (JSES Int 5:56, 2025).

**Aims:**

1: To propose an appropriate standard of care from the literature.

2: To compare a cohort of patients identified to the standard of care proposed.

**Methods:**

A mixed methodology study in a University Teaching Hospital. Quantitative research involving clinical audit and secondary analysis. Qualitative research involving in depth interviews with clinicians.

**Results:**

A cohort of 39 patients who presented between 1st January 2024 and 10th March 2025 were identified. Twenty six patients (67%) were female and 31 patients (79%) were aged over 50. Sixteen patients (41%) met the imaging protocol proposed by Favian et al. ^(3)^, while 36 patients (92%) achieved the management standard proposed by Wronka et al.^(4)^ In depth interview reiterated the need for CT prior to reduction and the importance of paralytic anaesthetic agents to reduce the risk of iatrogenic harm during certain reduction procedures.

**Conclusion:**

Patients aged over 50 or those with suspicion for occult neck of humerus fracture on plain film x-ray, should undergo CT prior to reduction. Fracture dislocations which are not isolated to the greater tuberosity should be referred to orthopaedics for consideration of reduction in theatre under general anaesthesia and a paralytic agent.

This manuscript has been prepared in the style of the Irish Journal of Medical Science.

## Background

Complicated shoulder dislocations involving an associated fracture, provide a clinical dilemma for emergency physicians due to the absence of guidelines or protocols both nationally and internationally. Isolated Greater Tuberosity (GT) fracture dislocations are routinely reduced in the emergency department (ED), but there is a risk of missing an occult neck of humerus fracture on x-ray [1]. Isolated GT fractures are relatively uncommon and account for 1% to 14% of proximal humerus fractures [2–4] and 57% of these occur in the setting of an anterior glenohumeral dislocation [2, 4].

Furthermore, there is known risk of iatrogenic harm when reducing a dislocated shoulder involving a fractured neck of humerus and this risk is higher without the use of an anaesthetic paralysing agent [5].

An extensive review of the literature revealed a paucity of evidence on two topics.

1: Criteria for which patients require CT imaging prior to reduction—while it is widely acknowledged that CT provides higher sensitivity imaging than x-ray [6], this is a finite resource and there is no protocol available to suggest which patients should be selected for CT.

Best available evidence: Favian et al. Level five, Senior author review [7].

2: The most appropriate method of reduction based on fracture type—while it is acknowledged that there is greater risk of iatrogenic harm from closed reduction under procedural sedation, no protocol exists to determine which patients can be safely managed without general anaesthesia and a paralytic agent.

Best available evidence: Wronka et al. Level three, retrospective cohort study [8].

Additionally, extensive discussion with senior trainers in both emergency medicine and upper limb orthopaedic surgery revealed an absence of protocols both nationally and internationally in the management of this condition.

A retrospective study by Yuan and Chua [5] outlined the increased risk of performing closed reduction under procedural sedation compared to general anaesthesia in theatre with the associated muscle relaxant effect. Although a management algorithm was proposed, this contained only level three evidence. While it is known that the majority of Greater Tuberosity fractures occur in the setting of a glenohumeral dislocation [2, 4] a study by Shaw et al. outlined the risk of both missed and occult surgical neck fractures when relying on plain film x-rays alone [1]. An imaging protocol proposed by Favian et al. [7] based on senior author review, outlined a cohort of patients who were at high risk of either occult or missed fractures to the neck of humerus based on x-ray alone, who should undergo CT prior to reduction as a standard of care. A study by Wronka et al. in 2017 [8] proposes a suitable management algorithm to decide which patients should be managed in the ED versus those patient who should be referred to the orthopaedic operating theatre.

## Aims

1: To propose an appropriate standard of care for the management of shoulder fracture dislocations, including mode of diagnostic imaging and method of reduction.

2: To examine the current management of patients with shoulder fracture dislocations presenting to the ED at St Vincent’s University Hospital (SVUH) and to compare to the proposed standard of care based on best available evidence.

## Methods

### Setting

St Vincent’s University Hospital (SVUH) is a trauma unit for the south east Dublin region, serving a population of over 300,000 with over 60,000 presentations every year, of which 25% are aged over 75. There are eight whole time equivalent (WTE) consultants in emergency medicine, one of whom provides an older persons rapid assessment hub (OPRAH).

### Overview

A mixed methodology study was deployed. As there is a paucity of research and evidence in the chosen topic, neither a quantitative nor qualitative study alone, was sufficient to answer this research question, making a mixed methods approach more appropriate [9].

### Subjects

- Quantitative research

A Cohort of 39 patients with shoulder fracture dislocations who presented between 1st January 2024 and 10th March 2025 were identified.

(b) Qualitative research

Clinicians from the specialties of emergency medicine and orthopaedics were selected for in depth interview. Level of seniority ranged from SHO to consultant.

### Data collection

- Quantitative research

Patients were identified using the picture archiving and communication system (PACS). This list was then cross referenced to both the orthopaedic operating list and the ED electronic record system (Maxims).

(b) Qualitative research

A questionnaire with 10 standardised topics (Appendix 1) was used. All meetings were recorded electronically and later transcribed using Apple voice memos.

### Analysis

- Quantitative research

The imaging protocol proposed by Favian et al. [7] was selected as the appropriate standard. Senior author opinion was that any patient over 50 years of age or with features suggestive of occult neck of humerus fracture on x-ray should undergo a CT prior to reduction. X-ray findings were assessed using retrospective radiology reports from PACS. Age at time of presentation, was assessed for using Maxims.

The management protocol proposed by Wronka et al. [8] was selected as the appropriate standard as they included both anterior and posterior dislocations. The suggested treatment by fracture dislocation type was as follows:

Isolated greater tuberosity fractures should have closed reduction attempted under sedation. Fractures to the surgical neck of humerus ± greater tuberosity should be managed with general anaesthesia and muscle relaxant ± fixation.

Any fracture associated with a posterior dislocation should be managed under general anaesthesia and muscle relaxant.

For the purposes of the study, fractures reported as “proximal humerus” and “head of humerus” were considered to involve a potential neck of humerus fracture as they extended further than an isolated greater tuberosity fracture. These were assessed as the second type of fracture in described by Wronka et al. [8]

(b) Qualitative research

Common themes were identified using the Braun and Clarke’s six thematic analysis [10].

### Ethical considerations

- Quantitative research

A retrospective clinical audit was approved by the SVUH clinical audit committee. Ethics approval for a secondary analysis of the primary audit was also provided by UCD School of Medicine UTMREC on 23rd May 2025, reference number UTMREC-SM-E-25–604-Slowey-Cullen. All patient identifiers were removed.

(b) Qualitative research

Ethics approval was provided by UCD School of Medicine UTMREC on 23rd May 2025, reference number UTMREC-SM-E-25–604-Slowey-Cullen. An invitation letter including an information leaflet (appendix 2) was emailed to clinicians. Consent forms were signed prior to the commencement of each meeting. Although the rank and specialty of the participant group been described, quotes remain anonymised to avoid identification by readers.

## Results

### Quantitative

Cases of shoulder fracture dislocations were identified over a 62 week period between 01/01/2024 and 10/03/2025, equating to 0.63 cases per week. Based on overall ED attendance of 79,756 for this time period, the incidence was 0.5% per 1,000 patients presenting to the ED.

**Fig. 1:**
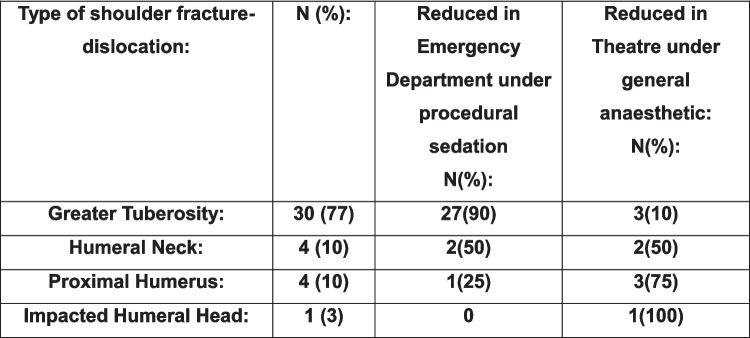
Management of fracture dislocation by fracture site

In total, 97% involved an anterior dislocation while one (3%) involved a posterior dislocation. Thirty of the 39 fractures (77%) were isolated to the GT (Fig. 1). Over all, 90% of the GT fracture dislocations were reduced in the ED under procedural sedation. Two of the four humeral neck fractures (50%) were reduced in the ED under procedural sedation while the other two were managed in theatre under general anaesthetic and a muscle relaxant. Three of the four proximal humerus fracture dislocations (75%) were managed in theatre as was the impacted head of humerus fracture dislocation (Fig. 1).

**Fig. 2:**
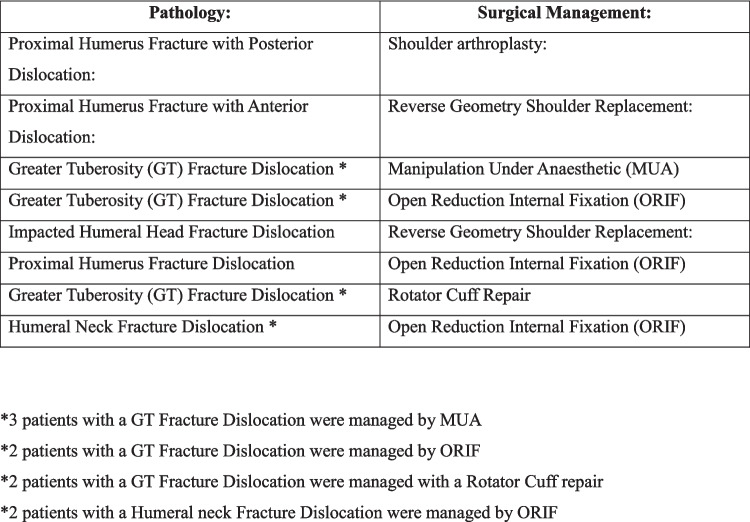
Surgical management by injury type. *3 patients with a GT Fracture Dislocation were managed by MUA. *2 patients with a GT Fracture Dislocation were managed by ORIF. *2 patients with a GT Fracture Dislocation were managed with a Rotator Cuff repair. *2 patients with a Humeral neck Fracture Dislocation were managed by ORIF

A total of 13 patients who presented with a fracture dislocation of the shoulder underwent surgery. One patient underwent surgery for a secondary rotator cuff injury, following initial reduction of a GT fracture dislocation in the ED. The remaining 12 patients underwent emergency surgery as part of their acute injury management (Fig. 2).

In total, 26 patients (67%) were female while 13 (33%) were male.

Over all, 23 injuries (59%) involved the left shoulder while 16 (41%) involved the right shoulder. No patient suffered bilateral shoulder fracture dislocations.

All four patients with a neck of humerus fracture were females aged over 50.

The ages of patients ranged from 15 to 89 with a mean age of 60 (SD = 19) a median age of 70 and a mode of 83.

**Fig. 3:**
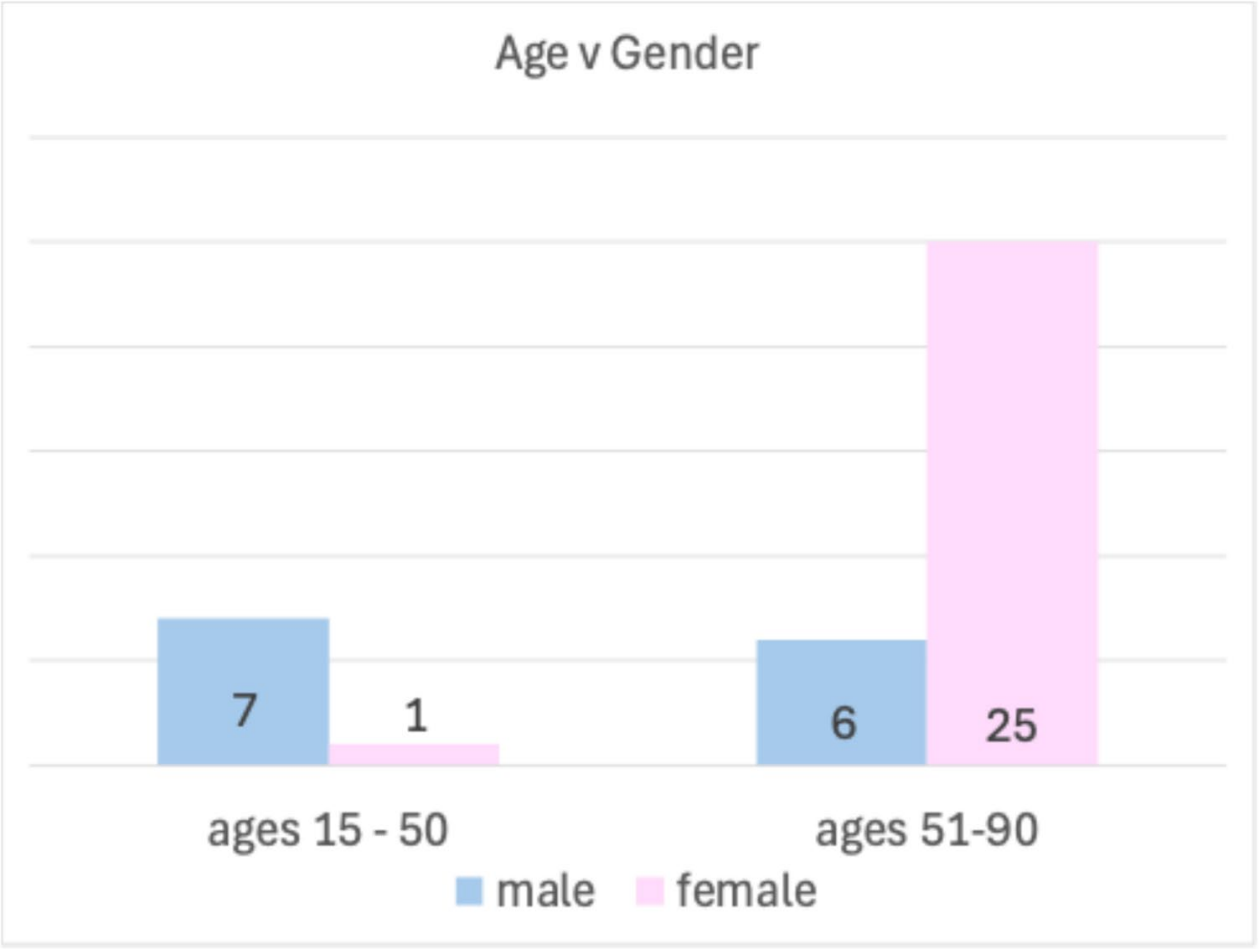
Gender breakdown by age

Over all, 31 patients (79%) were aged over 50 while eight (21%) were aged 50 and under. There was a greater dominance of females in the over 50 category and a dominance of males in the under 50 category. (Fig. 3) Although there is no variation in recommended management based on gender, it is important to note that 81% of patients aged over 50 with this condition were female. This has significant implications for bone health and further injury prevention. The existing literature suggests that post menopausal women are at higher risk for fragility fractures [11]. Post menopausal women who sustain this injury should be considered for screening of bone health, should they present with this injury.

In addition, thirty three patients (85%) presented to the ED out of hours, while six (15%) arrived during working hours. A total of 16 patients (41%) met the proposed imaging standard outlined by Favian et al. [7], while 23 patients (59%) did not meet this standard. From the patients who presented out of hours, 14 (42%) met the proposed imaging standard while 19 (58%) failed to do so. From six patients who presented during working hours, two (33%) met the imaging standard while four (67%) did not. The hypothesis proposed is that less experienced clinicians providing out of hours care in the absence of consultant presence may have a lower threshold to request a CT for this complex injury. Out of hours orthopaedic care is also provided through an on-call service which is off site. During working hours, multidisciplinary collaboration of both radiological imaging and clinical examination between emergency medicine and orthopaedics is available. Additionally, hot reporting of plain film x-ray is available through the radiologist during working hours while emergency physicians interpret x-ray images out of hours. These variations in working practices during different times of the week may reduce the perceived importance of CT, despite best evidence suggesting otherwise.

A total of 36 patients (92%) met the management standard proposed by Wronka et al. [8], while three patients (8%) failed to meet the standard. Of the 33 patients who presented out of hours, 30 patients (90%) met the proposed management standard. Of the six patients who presented during working hours all six patients (100%) were managed according to the proposed management standard. Of note, all three patients who failed to meet the management standard, presented out of hours.

Of the 12 patients managed in the operating room, five patients (42%) met the imaging standard proposed by Favian et al. [7], while seven (58%) failed to do so. Of the 27 patients managed in ED, 11 patients (41%) met the proposed imaging standard while 16 patients (59%) did not.

### Qualitative

A variable cohort of clinicians were recruited as illustrated in Table 1.

The following themes were identified using Braun and Clarke thematic analysis:

1: Uncomplicated shoulder dislocations are generally well managed:

2: Risk of iatrogenic harm when reducing fracture dislocations:

3: CT scan prior to reduction can help identify occult neck of humerus fractures:

Theme 1:

Uncomplicated shoulder dislocations are generally well managed:

All participants acknowledged that uncomplicated shoulder dislocations are well managed by emergency physicians in a safe and timely manner and are occasionally managed in theatre when difficulty arises.

One participant noted that:

*“I think generally shoulder dislocations are well managed. They all get reduced, whether it be with Penthrox initially or with procedural sedation or very occasionally ones where they have to go to theatre”*

Theme 2:

Risk of iatrogenic harm when reducing fracture dislocations:

Participants raised concerns regarding the known risks associated with reducing a fracture dislocation.

One participant noted:

*“If there’s a particular fracture across the neck of the humerus, there’s potential for the head of the humerus to end up in the axilla, with then the proximal end of the humerus being rammed into the glenoid fossa”*

Some participants had direct experience of adverse outcomes while others were aware of the theoretical risks.

Sub theme:

Absence of a protocol or guideline increases risk:

None of the participants were aware of a guideline for management, either nationally or internationally.

One participant noted:

*“Generally in emergency departments I don’t think that we follow what would be best evidence practice. There is no definitive protocol but there’s an increasing awareness of the difficulties, particularly in older patients with fracture dislocations that would need a CT prior to reduction”*

Participants noted that some fracture dislocations get referred directly to orthopaedics for reduction in theatre while some get reduced in the emergency department. This process is informal and unstructured with no clear rational or system for either approach.

Theme 3:

CT scan prior to reduction can help identify occult neck of humerus fractures:

All participants agreed that x-ray imaging lacks the sensitivity required to identify those patients with occult fractures of the neck of humerus.

One participant noted:

*“CT will be a really useful guidance, especially for showing any occult fractures that we weren’t aware of”*

All participants agreed that CT scan was their preferred mode of imaging prior to reduction.

Sub theme:

CT would help to identify patients more suitable for reduction under general anaesthesia:

Participants agreed that they would refer high risk patients to orthopaedics for reduction based on positive CT findings.

One participant noted:

*“They would give paralytic agents like rocuronium to establish more of a muscle relaxation so a safe reduction can be done, if not an open reduction”*

This reiterated the value of CT prior to reduction where possible.

## Discussion

### Key findings

Findings from the quantitative data show that shoulder fracture dislocations are an injury of the older adult, with 31 out of 39 patients (79%) aged over 50. The mean age was 60 and the median age 70. However, the qualitative interviews suggest that elderly patients are the ones at risk of iatrogenic harm due to fracture propagation. Additionally, 25 of 31 patients (81%) aged over 50 with this injury were female, while all four patients (100%) with a confirmed neck of humerus fracture were females aged over 50.

Although there were no adverse outcomes observed in the patient population studied, 59% of patients aged over 50 did not undergo a CT prior to reduction. This shows potential for harm in 23 patients over a 62 week period. Additionally, two neck of humerus fractures and one proximal humerus fracture were reduced in the ED under procedural sedation, which shows additional potential for harm in these three patients.

The majority (85%) of patients presented out of hours, which highlights the need for a robust guideline at times when the most senior decision makers may be off site. Interestingly, a higher proportion of patients presenting out of hours (74%) received appropriate imaging prior to reduction compared to 50% of patients who presented during working hours. This suggests that absence of clear guidance was a significant factor rather than access to CT. While 33 patients (85%) presented out of hours, 30 of these (90%) received the appropriate management, which suggests that resources and access to theatre is not a significant barrier.

### Comparison with the existing literature

The higher fracture dislocation rates observed in older women are consistent with the existing literature suggesting that the prevalence of osteoporosis and rate of fractures are much higher in post menopausal women than in older men [11].

A retrospective study by Yuan et al. showed a similar age profile of injury, with mean age of 62 compared with a mean age of 60 in this study [5]. Favian et al. [7] identified female sex and age greater than 50 as risk factors for an occult for an occult neck of humerus fracture. This is consistent with findings in this study, where all four patients (100%) with an associated neck of humerus fracture were female and aged over 50.

### Methodological considerations

To the author’s knowledge, this is the first study of shoulder fracture dislocations in the Republic of Ireland. This study has a number of potential limitations:

X-Ray findings on presentation were based on retrospective radiology reports rather than clinician interpretation at the time of assessment.

SVUH has an older catchment area which may have biased the cohort studied. Over 25% of patients attending SVUH are aged over 75 years.

Patients with fractures to the humeral head or proximal humerus were not accounted for in the management algorithm proposed by Wronka et al. [8]. Four of these five cases (80%) were managed in theatre under general anaesthesia, suggesting that they are significant injuries that need to be considered in any further studies.

### Implications for research and practice

This study highlights the need for further, multicentred research. There is scope for the development of an expert panel within RCSI between emergency medicine and orthopaedics, to develop a national protocol for shoulder dislocations and an associated management algorithm for shoulder fracture dislocations. Larger scale, multi centre retrospective audit would be a valuable tool to inform such a policy.

Based on the results of this single centre study along with the existing evidence, a proposed algorithm may include the following:

1: Imaging protocol as described by Favian et al. [7]

2: Management protocol as described:

(a): An anterior shoulder fracture-dislocation isolated to the greater tuberosity should undergo attempted reduction under procedural sedation in the emergency department.

If reduction attempts fail, referral to the orthopaedic team on call to consider manipulation under anaesthesia (MUA) or open reduction internal fixation (ORIF) under general anaesthesia and a paralytic agent.

(b): An anterior shoulder fracture-dislocation involving more than the greater tuberosity should be referred to the orthopaedic team on call for MUA or ORIF under general anaesthesia and a paralytic agent.

(c): Any posterior shoulder fracture-dislocation should be referred to the orthopaedic team on call for MUA or ORIF under general anaesthesia and a paralytic agent.

**Table 1:** Clinicians interviewed

Rank:	Specialty:	Special Interest:
Consultant	Emergency Medicine	Sports & Exercise Medicine
Consultant	Orthopaedics	Upper Limb
Consultant	Emergency Medicine	Ambulatory Care
SPR	Emergency Medicine	Sports & Exercise Medicine
Registrar	Emergency Medicine	Trauma
SHO	Orthopaedics	Trauma

## Appendix 1

### Qualitative interview questionnaire

**Table:**

**Question 1:**	**What is your perception of the current management of shoulder dislocations?**
Question 2:	**Are you aware of any protocol regarding shoulder dislocations in your institution?**
Question 3:	**What management strategies are you aware of regarding fracture-dislocations of the shoulder?**
Question 4:	**Are there any potential adverse events regarding the reduction of shoulder fracture-dislocations that would concern you?**
Question 5:	**Is there any evidence to suggest how fracture-dislocations should be managed according to best practice?**
Question 6:	**Are fracture-dislocations of the shoulder a common occurrence?**
Question 7:	**How are fracture—dislocations of the shoulder diagnosed radiologically?**
Question 8:	**Are there any red flags on radiological imaging that clinicians commonly look for?**
Question 9:	**Are there any potential ways that radiological imaging could best inform the management of fracture-dislocations of the shoulder?**
Question 10:	**Would the introduction of a guideline, either nationally or locally assist with the management of fracture-dislocations of the shoulder?**

## Appendix 2

### Information leaflet provided

Dear Doctor.

Thank you for expressing interest in our qualitative research into the management of fracture-dislocations of the shoulder, presenting to the emergency department.

This area of research has been conducted in association with the UCD school of Medicine, MSc in Primary Care (Orthopaedic & Musculoskeletal Medicine), under the supervision of Prof Walter Cullen.

We aim to conduct interviews, which will be facilitated at a time of your choice and convenience. Interviews will be recorded for data collection. However, all data collected will be fully anonymised and recordings will be destroyed following the completion of data collection. While we will disclose the specialty and rank of interviewees as part of our research, we will not disclose any personal details.

All ten questions asked will be open ended and we value your opinion on all matters relating to this topic. Questions will not relate to specific patient cases and will not require disclosure of personal experiences. Rather, we hope to gather your personal opinion on the current practice in this area based on your professional experience.

I have attached a consent form, which you may choose to return at your convenience. Additionally, you may specific a date and time that you would be available to participate in this project.

Yours sincerely.

Dr Joseph Slowey.

Post CCST Fellow in Emergency Medicine.

MSc candidate in Orthopaedics and Musculoskeletal Medicine.
